# The Secretion from Neural Stem Cells Pretreated with Lycopene Protects against *tert*-Butyl Hydroperoxide-Induced Neuron Oxidative Damage

**DOI:** 10.1155/2018/5490218

**Published:** 2018-07-22

**Authors:** Cuiqin Huang, Danhui Gan, Chongzhu Fan, Caiyan Wen, An Li, Qin Li, Jiayi Zhao, Zhen Wang, Lihong Zhu, Daxiang Lu

**Affiliations:** ^1^Department of Pathophysiology, Institute of Brain Science Research, Key Laboratory of State Administration of Traditional Chinese Medicine of the People's Republic of China, School of Medicine, Jinan University, Guangzhou, Guangdong 510632, China; ^2^Department of Pathology, Guangzhou Overseas Chinese Hospital, The First Affiliated Hospital of Jinan University, Guangzhou, Guangdong 510630, China

## Abstract

Neural stem cells (NSCs) hold great potential for the treatment of Alzheimer's disease (AD) through both cellular replacement and their secretion of trophic factors. Lycopene is a potent *β*-carotenoid antioxidant that has been shown to ameliorate oxidative damage in previous studies. However, it is unclear if lycopene can interact with NSCs to induce the secretion of growth factors, and whether pretreatment with lycopene will allow NSCs to secrete enough trophic factors to reduce oxidative damage to neurons. We pretreated cultured NSCs with lycopene, then applied the lycopene-treated-NSC-conditioned media (Ly-NSC-CM) to primary neuronal cultures exposed to *tert*-butyl hydroperoxide (t-BHP) to induce oxidative damage. We found that lycopene promoted the secretion of nerve growth factor (NGF), brain-derived neurotrophic factor (BDNF), and vascular endothelial growth factor (VEGF) from NSCs. In addition, Ly-NSC-CM attenuated oxidative stress and reduced t-BHP-induced cell apoptosis. We found an antiapoptotic effect related to inhibited expression of Bax/Bcl-2, cytochrome C, and cleaved caspase-3. Moreover, Ly-NSC-CM increased the levels of synaptic proteins, including synaptophysin (SYP) and postsynaptic density 95 (PSD-95), and activated the PI3K/Akt pathway in cultured neurons. Collectively, these data indicate that Ly-NSC-CM could protect neurons from t-BHP-induced oxidative damage.

## 1. Introduction

Alzheimer's disease (AD) is a common neurodegenerative disease worldwide. AD manifests as impaired cognition and memory, with dramatically increasing morbidity as a consequence of aging. Extracellular and intracellular amyloid-beta (A*β*) deposits are known to be a key pathologic characteristic of AD [1–4]. Previous studies revealed that A*β* also interacts with mitochondria, resulting in mitochondrial dysfunction and increased oxidative stress [5–8] from reactive oxygen species (ROS). Excess mitochondrial ROS production resulting from age and mitochondrial dysfunction, which occurs in AD, damages proteins and DNA; further, when ROS are released after apoptosis, they damage other cells leading to a cytotoxic domino effect [9, 10]. Moreover, ROS cause disturbances in neurotransmission and gradually increase the risk of developing AD [11]. Experimentally, one method of artificially producing ROS is exposure to *tert*-butyl hydroperoxide (t-BHP), a stable peroxide that produces a large amount of free radicals and induces oxidative stress and its responses (mitochondrial dysfunction and apoptosis) in vitro [12]. In fact, our previous study demonstrated that t-BHP induces amyloid aggregation in cultured neurons [13].

Neural stem cells (NSCs) are self-renewing precursors of neurons, astrocytes, and oligodendrocytes; common during fetal development, they also reside in specialized niches such as the subventricular zone (SVZ) and the subgranular zone (SGZ) in the adult central nervous system (CNS). Compelling evidence has established that neural stem cells (NSCs) exert a substantial beneficial and therapeutic effect after transplantation in experimental neurodegeneration disease models. NSCs repair damaged tissue not only by physically replacing cells but also by secreting neurotrophic or immune modulatory factors [14, 15]. In oxygen-deprived conditions, NSCs constitutively produce and secrete neurotrophic factors and hormones, such as nerve growth factor (NGF), brain-derived neurotrophic factor (BDNF), and vascular endothelial growth factor (VEGF). These factors form a complex bioactive network locally and improve the function and structure of the target tissues [16, 17]. Experimental evidence has been published confirming the neuroprotective effects of NSC-secreted factors in AD [18, 19]. Furthermore, it has been reported that cytokine preconditioning augments secretion of trophic factors by NSCs [20]. For example, in mice with induced ischemic brain damage, preconditioning with IL-6 prior to cell transplantation enhanced the therapeutic effects of NSCs by upregulating mitochondrial antioxidant enzymes and VEGF secretion [20].

In terms of clinical application, NSC secretion may have significant advantages over the use of the NSCs themselves. Secreted NSCs can bypass immunological compatibility problems associated with cell therapy, the disease itself, and other related problems and can also reduce the time and cost of cell culture and maintenance. Since secreted NSCs can be mass-produced, stored for long periods, and reused (unlike implanted cells), they provide an advantage of much more cost-efficient treatment. NSC-conditioned medium could therefore be used in regenerative medicine to provide a significantly greater therapeutic benefit from the same pool of exogenous NSCs.

Lycopene is a carotenoid primarily found in tomatoes and other red fruits that is an extremely potent antioxidant, more so even than beta-carotene. Lycopene has shown diverse neuroprotective bioactivities against inflammation, cancer, and oxidative damage. Whether these properties have therapeutic benefits in AD has begun to be examined recently. Thus far, lycopene is reported to significantly reduce amyloid-induced neurotoxicity in cultured neurons and to ameliorate memory deficits and mitochondrial oxidative stress in A*β*1–42-injected rats [21–23]. However, the effects of lycopene on both the secretion of cytokines and neurotrophic factors by NSCs and the neuroprotective capacity of the conditioned medium are unknown. This study sought to determine the effects of lycopene pretreatment on NSC secretion and the neuroprotective capacity of the conditioned medium, as well as to explore the putative mechanisms for the observed effects.

## 2. Materials and Methods

### 2.1. Primary Cultures of Hippocampal NSCs and Cerebral Cortical Neurons

C57BL/6J mice were obtained from the Animal Experiment Center of Southern Medical University (License number 44002100007907) and were used within 24 hours after birth. All the procedures were approved by the Ethics Committee of the Institute of Laboratory Animal Science, Jinan University. Primary cerebral cortical neurons were cultured as described previously [24, 25] with some revisions. Briefly, the neocortex and hippocampus were dissected from neonatal C57BL/6J mice, and meninges and blood vessels were dissociated from the cells in HBSS (Gibco). The hippocampal cells were resuspended in Dulbecco's modified Eagle's medium (DMEM) and F12 (1 : 1; D/F; Gibco) to isolate the neurons, then plated in a 25 cm^2^ cell culture flask at 2 × 10^5^ cells/mL in D/F containing 2% B27 supplement (Gibco). If the media were supporting NSCs, 20 ng/mL bFGF (Sigma) and 20 ng/mL EGF (Sigma) were added to the D/F in addition to B27. The cells were cultured at 37°C with 5% CO_2_ and supplied with complete medium (primary D/F: supply D/F = 1 : 1) every three days [25]; at the same time, neurospheres were separated by centrifuging at 650*g* for 5 min, and then the primary medium was collected after further centrifuging at 3500*g* for 5 min. Half of the medium was changed every 3 days. Cultures were used for experiments on day 9.

The neocortex was cut into small pieces, dissociated using 0.125% trypsin (Gibco) for 20 min at 37°C, and filtered through a cell strainer (Gibco). Neocortical cells were obtained after centrifugation at 1000*g* for 5 min and plated on poly-D-lysine-coated culture plates in D/F containing 10% fetal bovine serum (FBS; Gibco) and 50 mg/mL streptomycin/penicillin (Hyclone). The cells were maintained in an incubator at 37°C under 5% CO_2_/95% O_2_. After 4 hours, the medium was replaced with Neurobasal A medium (Gibco) containing 2% B27 supplement. Half of the medium was replaced every 3 days. Cultures were used for in vitro experiments at day 7. To evaluate the morphology of neurons and NSCs, cells were observed using a microscope (Leica) with phase-contrast optics.

### 2.2. Cell Treatment

Lycopene was purchased from the National Institute for the Control of Pharmaceutical and Biological Products (Guangzhou, China) and was dissolved in tetrahydrofuran (THF) including 0.025% butylated hydroxytoluene (BHT) to stop the formation of peroxides. The stock solution was protected from light and humidity and stored at −80°C. Before each experiment, the stock solution of THF-lycopene was diluted in D/F to the final concentrations to be applied. The amount of THF vehicle in the culture medium was never greater than 0.1% (*v/v*), a concentration that has been shown not to affect the assays (results were similar to application of vehicle-free control medium) [26]. D/F was used as the vehicle control medium. *tert*-BHP (t-BHP) was purchased from Aladdin (China), diluted in D/F, and stored at 4°C. Groups of neurons were first assigned to six concentration groups (0 *μ*M, 2.5 *μ*M, 5 *μ*M, 10 *μ*M, 20 *μ*M, and 40 *μ*M t-BHP) and exposed for 24 h, to identify a working dose. Neurobasal A was used as a vehicle control medium for these primary neuronal cultures. NSCs were preconditioned with different concentrations (0 *μ*M, 0.1 *μ*M, 1 *μ*M, 2 *μ*M, 4 *μ*M, 8 *μ*M, and 16 *μ*M) of lycopene for 24 h, after which the media (NSC-conditioned media) were collected. Neurons were pretreated with NSC-conditioned media for 4 h and then exposed to t-BHP for 24 h in each individual experiment.

### 2.3. Cell Viability Assay

**Figure 1:**
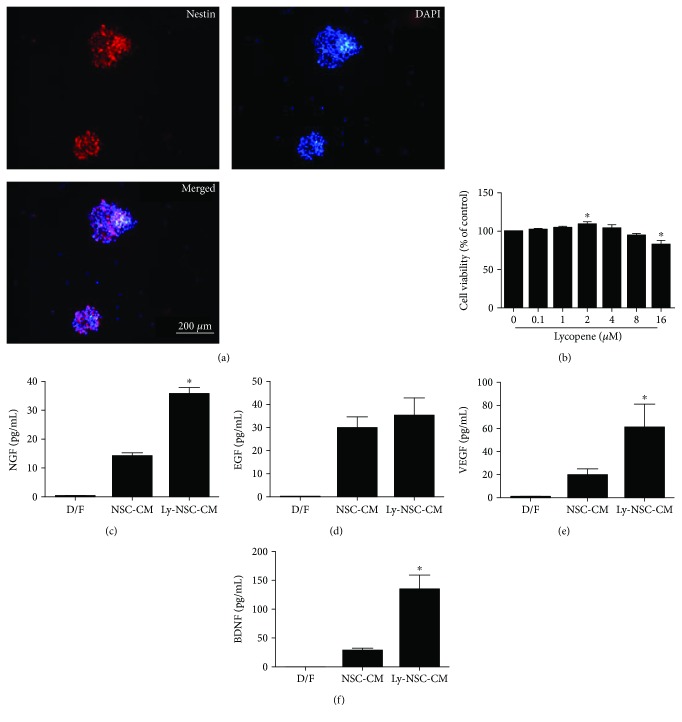
The effects of lycopene on neural stem cells (NSCs). (a) NSC identification: nestin protein expressed in cultured NSCs and visualized by immunofluorescence. Red (TRITC-labeled) nestin immunoreactivity was observed in the peripheral cytoplasm of NSCs; all nuclei are stained with DAPI (blue). (b) NSCs were treated with lycopene doses from 0.1 *μ*M to 16 *μ*M for 24 hours, and cell viability was examined by MTT assay. (c–f) NSCs were pretreated with the working dose of 2 *μ*M lycopene or D/F vehicle for 24 hours. The levels of NGF, EGF, BDNF, and VEGF were measured by ELISA. *n* = 3 per group. Data was described as mean ± SEM. ^∗^*P* < 0.05 versus NSC-CM group.

**Figure 2:**
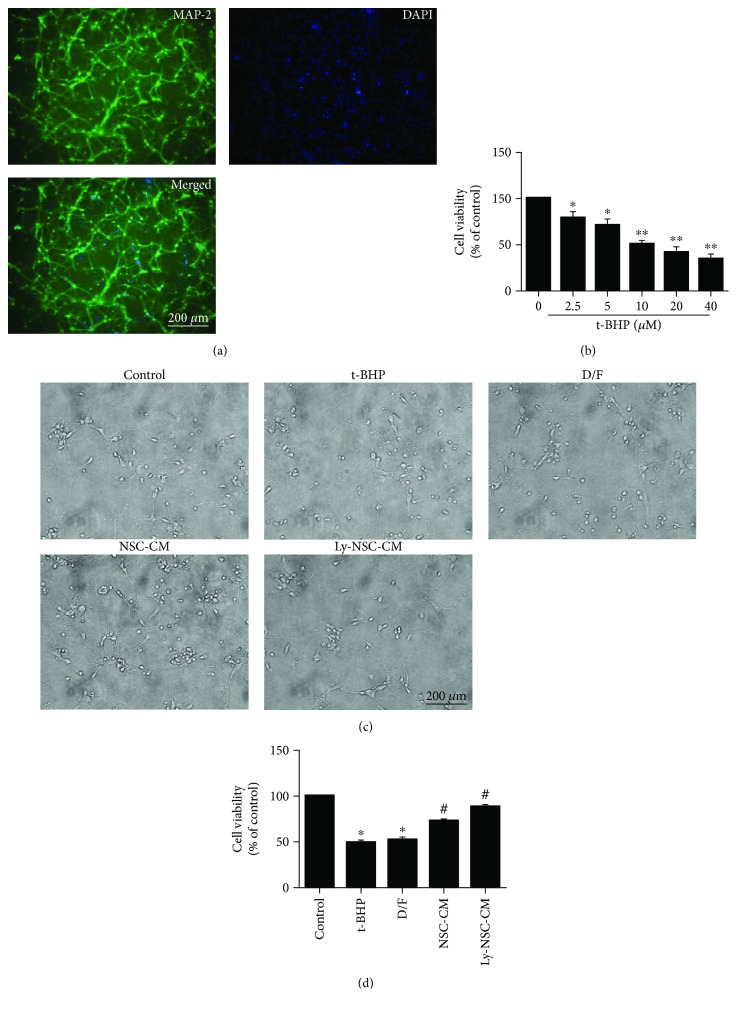
The effects of Ly-NSC-CM treatment on neuronal damage. (a) Neocortical neuron identification: analysis of MAP-2 (green) protein immunofluorescence in mouse primary neocortical neuron cultures. (b) Neurons were treated with t-BHP from 2.5 *μ*M to 40 *μ*M for 24 h, and cell viability was examined by MTT assay. (c) The morphology of neocortical neurons and their neurite extensions. (d) Neuronal cell viability according to an MTT assay. *n* = 3 per group. Data are described as mean ± SEM. ^∗^*P* < 0.05 versus control group. ^#^*P* < 0.05 versus t-BHP group.

Cell viability was assessed using the 3-(4,5-dimethylthiazol-2-yl)-2,5-diphenyltetrazolium bromide (MTT) method according to the manufacturer's instructions (Beyotime, China). Briefly, NSCs or neurons cultured in 96-well plates were treated as described above in Cell Treatment (see also Figures 1(b), 2(b), and 2(d)), then incubated for an additional 4 h at 37°C after adding 5 mg/mL MTT to the medium already in the plate. The MTT solution was removed, and the colored formazan crystals were dissolved in 150 *μ*L dimethylsulfoxide. The optical density (OD) values were measured using an iMark Microplate Absorbance Reader (Bio-Rad) at 570 nm. Cell viability was expressed as the ratio of the signal obtained from the treated group to that from the control group in percent form (i.e., the control group = 100%).

### 2.4. NSC Secretion (Conditioned Medium) Collection

Mouse primary hippocampal NSCs were seeded in 25 cm^2^ culture flasks (2 × 10^5^ cells/mL) and, upon reaching 80% confluency, were treated with 2 *μ*M lycopene diluted from the stock described in “Cell Treatment” for 24 h. They were then gently washed three times with phosphate-buffered saline (PBS, Gibco), and the medium was replaced with fresh D/F. This (conditioned) culture medium was decanted from the NSCs 6 h later and then centrifuged at 1610*g* for 5 min. The supernatants were stored at 4°C before being used experimentally (control NSC-conditioned medium, NSC-CM; lycopene-treated-NSC-conditioned, Ly-NSC-CM). Treatment media were diluted with Neurobasal A medium for use in vitro.

### 2.5. ELISA of Conditioned Media

NSC-CM and Ly-NSC-CM media were tested without dilution for levels of NGF, epidermal growth factor (EGF), BDNF, insulin-like growth factor 1 (IGF-1), basic fibroblast growth factor (bFGF), and VEGF using the appropriate ELISA (CUSABIO, Wuhan, China) according to manufacturer's instructions.

### 2.6. Measurement of Intracellular ROS

The dichlorofluorescein diacetate assay (DCFH-DA, Beyotime, Nanjing, China) was used to determine the level of intracellular ROS. In brief, DCFH-DA was diluted just prior to use with D/F to a 10 *μ*M working concentration. Mouse primary cortical neurons were seeded on six-well plates and cultured for 7 days, then treated with one of the conditioned media (see NSC Secretion (Conditioned Medium) Collection). After each treatment, the conditioned medium was removed and the neurons were washed with PBS; they were then incubated with DCFH-DA (10 *μ*M) in the dark for 20 min at 37°C. After DCFH-DA incubation, neurons were washed as before and collected. A BD FACSAria flow cytometer (BD Biosciences, USA) was used to measure the fluorescent intensity changes (intracellular ROS) at a detection wavelength of 488 nm.

### 2.7. Determination of Mitochondrial Membrane Potential (ΔΨm)

The ΔΨm was measured with the JC-1 Mitochondrial Membrane Potential Assay kit (Beyotime, Shanghai, China). Primary cortical neurons were plated onto poly-D-lysine-coated six-well plates and cultured for 7 days, then treated with one of the conditioned media. After each treatment, neurons were treated with JC-1 at 37°C for 30 min according to the manufacturer's instructions, and representative images were obtained using a fluorescence microscope. The fluorescence peaks of monomers (green, 534 nm) and aggregates (red, 594 nm) were analyzed. The ratio of red to green fluorescence was used to quantify the ΔΨm of the cortical neurons in each treatment group, such that a larger red : green ratio represents a more polarized, intact mitochondrial membrane.

### 2.8. Immunofluorescence

Primary NSCs and neurons were plated on poly-D-lysine-coated six-well plate for 7 days. After conditioned medium treatment, the medium was decanted and the cells were washed three times with PBS; they were then fixed with 4% paraformaldehyde for 30 min. Then NSCs and neurons were permeabilized with 0.3% Triton X-100 in PBS for 15 min. After being blocked in 3% bovine serum albumin (BSA) with PBS at room temperature for 1 h, NSCs and neurons were washed again in PBS and finally incubated overnight at 4°C with one of anti-MAP 2, anti-nestin, or anti-cleaved caspase-3 antibodies (1 : 200, Cell Signaling Technology, USA). Cells were then washed with PBS and incubated in one of two secondary antibodies (TRITC-anti-rabbit (555 nm) and DyLight 488-anti-rabbit 1 : 400, Cell Signaling Technology, USA) for 2 h. A 10 min incubation in DAPI was used to counterstain nuclei. Finally, fluorescent images were captured on an epifluorescence microscope (Leica, Germany), and fluorescence intensity was measured with ImageJ software.

### 2.9. TUNEL Assay

The terminal deoxynucleotidyl transferase-mediated biotinylated UTP nick-end labeling (TUNEL) assay was performed to identify apoptotic cells using an in situ cell death detection (POD) kit (KeyGEN BioTECH, Jiangsu, China). After conditioned medium treatment, neurons cultured on coverslips in poly-D-lysine-coated six-well plates for 7 days were rinsed three times with PBS and fixed with freshly prepared 4% paraformaldehyde for 20 min at room temperature. Cells were blocked in 3% H_2_O_2_ with PBS for 15 min and then permeabilized in ice-cold 0.1% Triton X-100 with PBS for 10 min. TUNEL-positive (apoptotic) neurons were identified and counted according to the assay manufacturer's instructions. Nuclei were counterstained with 4′,6-diamidino-2-phenylindole (DAPI, Beyotime, Nantong, Jiangsu, China), and the number of TUNEL^+^-/DAPI^+^-neurons converted to a percentage of the total DAPI^+^-nuclei in four nonoverlapping fields per coverslip.

### 2.10. Western Blot Analysis

After the conditioned medium treatment, neurons were harvested and lysed on ice in cell lysis buffer (Beyotime, Nantong, Jiangsu, China) for 30 min. The total cellular proteins were centrifuged at 4°C and 11,600*g* for 20 min, and the protein concentration was measured using the enhanced BCA Protein assay kit (Beyotime, Jiangsu, China). Equal amounts of proteins were separated by 12% SDS-PAGE (Beyotime, Jiangsu, China) for quantifying GAPDH, cytochrome C, caspase-3, cleaved caspase-3, Bax, and Bcl-2 and 8% SDS-PAGE for quantifying *β*-tubulin, SYP, PSD95, PI3K, p-PI3K, Akt, and p-Akt. The separated proteins were then electrically transferred to a polyvinylidene fluoride membrane (PVDF, Millipore, USA). The membranes were blocked for 1 hour in 5% nonfat dry milk (Beyotime, Jiangsu, China) in Tris-buffered saline with 0.1% Tween 20 (TBST). After being washed with TBST, the membranes were incubated with the appropriate primary antibodies for the proteins listed above (all rabbit, 1 : 1000, Cell Signaling Technology, USA) at 4°C overnight. After washing with TBST, the membranes were incubated for 1 h in a horseradish peroxidase-conjugated secondary antibody (anti-rabbit, 1 : 5000, Cell Signaling Technology, USA). Signals were measured with an enhanced chemiluminescence kit (ECL, Millipore, USA) on a gel imaging system (Millipore, Billerica, MA, USA), and the results were visualized using Quantity One software.

### 2.11. Assessment of Cytochrome C Release

Primary mouse cerebrocortical neurons were collected and washed with PBS to prepare the cytosolic fraction after each treatment. The cell was resuspended in 500 *μ*L buffer A (pH 7.5, 1.5 mM MgCl_2_, 20 mM HEPES-KOH, 10 mM KCl, 1 mM sodium EDTA, 1 mM leupeptin, and 1 *μ*g/mL chymostatin) and homogenized using a Pyrex glass homogenizer and a type B pestle (40 strokes). The homogenate was centrifuged at 4°C, 11,600*g* for 20 min to generate the cytosolic fraction, and these supernatants (protein) were collected for Western Blot Analysis using a monoclonal antibody against cytochrome C.

### 2.12. Statistical Analysis

All numerical data are presented as mean ± SEM (*n* > 3). One-way analysis of variance (ANOVA) and Student's *t*-test, as appropriate, were used to analyze significant differences between groups. Differences with a *P* < 0.05 were considered to be significant. GraphPad Prism 5.3 (GraphPad Software, San Diego, CA) was used to generate all graphs.

## 3. Results

### 3.1. Lycopene-Induced Secretion of NGF, VEGF, and BDNF by NSCs

First, hippocampal NSCs were identified by an analysis of nestin protein expression using immunofluorescence (Figure 1(a)). The NSCs were exposed to a range of lycopene concentrations (0.1 *μ*M to 16 *μ*M) for 24 h to determine a working dose (Figure 1(b)). Cell viability was determined by MTT. We found that 2 *μ*M lycopene treatment demonstrated the largest enhancement of cell viability and a significant reduction in viability at 16 *μ*M. Hence, the supernatants collected as Ly-NSC-CM represent pretreatment with 2 *μ*M lycopene or D/F (vehicle) pretreatment (NSC-CM), with D/F medium without pretreatment also serving as a control. We found that the levels of NGF, VEGF, and BDNF in Ly-NSC-CM were significantly higher than those in the NSC-CM and D/F (*P* < 0.05; Figures 1(c), 1(e), and 1(f)) groups. EGF was present but without significant differences between groups (*P* > 0.05; Figure 1(d)), whereas bFGF and IGF-1 were not detected.

### 3.2. Ly-NSC-CM Increased Cell Viability and Reduced Neurite Damage in t-BHP-Induced Neurons

We used MAP-2 immunoreactivity to identify neocortical neurons (Figure 2(a)), then exposed them to a range of t-BHP concentrations (2.5 *μ*M to 40 *μ*M) for 24 hours to determine a working dose. Cell viability was then assessed by MTT assay. t-BHP evoked a dose-dependent decrease in the viability of cultured neurons (Figure 2(b)). Neurons were pretreated with NSC-CM and Ly-NSC-CM for 4 hours before being treated with 10 *μ*M t-BHP for 24 hours. Morphological changes associated with t-BHP toxicity were assessed qualitatively by phase-contrast microscopy (Figure 2(c)). Many neurites had extended to form anatomical networks in control cultures. However, in the t-BHP-treated neurons, neuronal cell bodies were smaller and the neurites were shorter or no longer visible. The t-BHP-induced morphological alterations appeared to be prevented by pretreatment with Ly-NSC-CM. Cell viability was significantly decreased by 50% after 10 *μ*M t-BHP exposure (*P* < 0.05; Figure 2(d)), while cell viability in the NSC-CM- and Ly-NSC-CM-pretreated groups were increased to 73% and 88% of vehicle-treated controls, respectively (*P* < 0.05; Figure 2(d)). These results suggest that lycopene-induced NSC secretion may enhance neuronal survival and reduce oxidative damage.

### 3.3. Ly-NSC-CM Suppressed Intracellular ROS Generation in t-BHP-Treated Neurons

**Figure 3:**
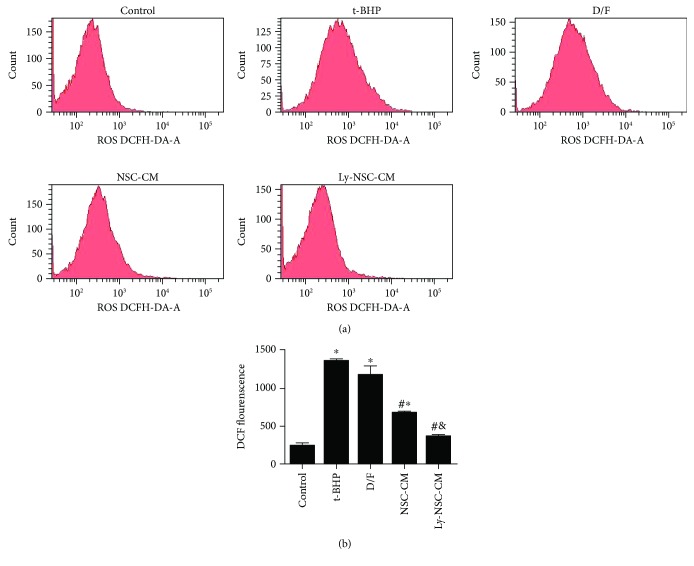
Reactive oxygen species (ROS) production in cultured primary neocortical neurons. (a) Neurons were pretreated with NSC-CM and Ly-NSC-CM for 4 hours, then exposed to t-BHP (10 *μ*M) for 24 hours. The generation of ROS was detected by flow cytometry. (b) The ROS levels were assayed by measuring the fluorescence intensity of DCF. Mean ± SEM. *n* = 3 per group. ^∗^*P* < 0.05 versus control group. ^#^*P* < 0.05 versus t-BHP group. ^&^*P* < 0.05 versus NSC-CM group.

The induction of oxidative stress by excess intracellular ROS generation is widely accepted as one likely upstream mechanisms of t-BHP-induced neurotoxicity. The intracellular ROS level was measured by DCF fluorometry during exposure to 10 *μ*M t-BHP for 24 hours. The production of ROS (Figure 3(a)) was significantly higher than controls in t-BHP-induced neurons (approximately 573% of the control group, *P* < 0.05). NSC-CM and Ly-NSC-CM pretreatments reduced t-BHP-induced ROS generation significantly, to approximately 286% and 108% of the control group, respectively (*P* < 0.05; Figure 3(b)). Ly-NSC-CM inhibited t-BHP-induced ROS generation significantly more than did NSC-CM (*P* < 0.05; Figure 3(b)).

### 3.4. Ly-NSC-CM Inhibited the Loss of ΔΨm in t-BHP-Treated Neurons

**Figure 4:**
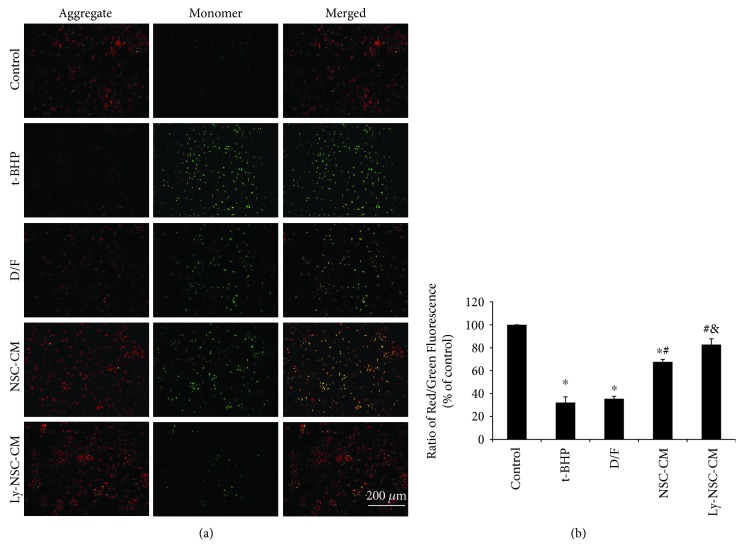
Effect of indicated treatment on the mitochondrial membrane potential (ΔΨm) in cultured neurons. (a) Representative images of neurons stained by JC-1. Red fluorescence represents JC-1 aggregates in healthy (polarized) mitochondria, whereas green fluorescence represents cytosolic JC-1 monomers released from mitochondria with depolarized membranes. (b) The ΔΨm of neurons in each group was calculated as the fluorescence ratio of red to green. Mean ± SEM. *n* = 3 per group. ^∗^*P* < 0.05 versus control group. ^#^*P* < 0.05 versus t-BHP group. ^&^*P* < 0.05 versus NSC-CM group.

Depolarization of the mitochondrial membrane potential (ΔΨm) is a critical event in the mitochondrial pathway of inducing apoptosis, which is generally related to oxidative stress. Relative to the control group, exposure to 10 *μ*M t-BHP for 24 hours evoked a significant reduction (depolarization of the membrane) in the initial ΔΨm, based on the decreased JC-1 fluorescence at 596 nm (red) and concomitantly increased fluorescence at 534 nm (green). After Ly-NSC-CM pretreatment for 4 hours, a significant restoration of ΔΨm was observed (Figure 4(a)). The red : green fluorescence ratio was 32% lower in the t-BHP group than in the control group (*P* < 0.05; Figure 4(b)); both NSC-CM and Ly-NSC-CM pretreatment significantly accelerated the restoration of ΔΨm (*P* < 0.05; Figure 4(b)). Ly-NSC-CM was significantly more effective than NSC-CM as well (*P* < 0.05; Figure 4(b)).

### 3.5. Ly-NSC-CM Attenuated t-BHP-Induced Apoptosis in Neocortical Neurons

**Figure 5:**
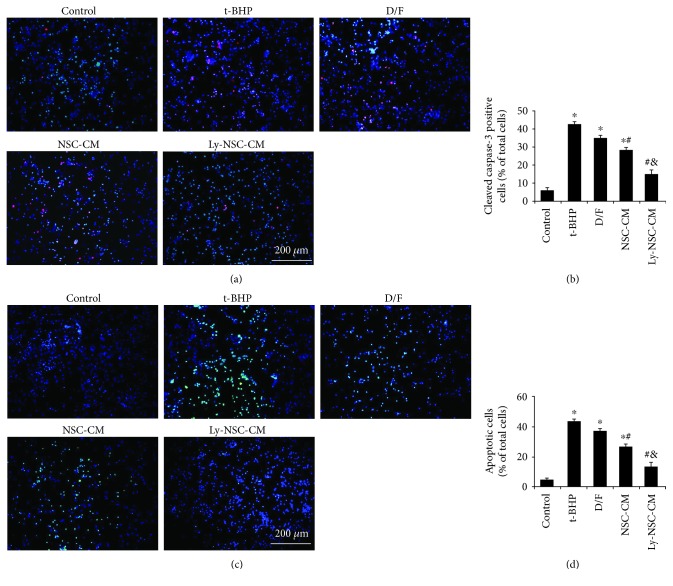
Ly-NSC-CM protects cultured neurons against t-BHP-induced apoptosis. (a) Immunofluorescence imaging of neurons labeled with cleaved caspase-3 (red) and DAPI (blue). (b) Quantification of cleaved caspase-3 labeling. The histogram displays the proportion of cleaved caspase-3-positive cells (as a % of total cells) in each group. (c) Representative images of TUNEL-positive cells (green) and DAPI (blue). (d) Quantification of the TUNEL assay. The histogram displays the percentage of TUNEL-positive cells (as % of total cells) in each group. *n* = 3. Data are described as mean ± SEM. ^∗^*P* < 0.05 versus control group. ^#^*P* < 0.05 versus t-BHP group. ^&^*P* < 0.05 versus NSC-CM group.

To further investigate whether Ly-NSC-CM can influence cell apoptosis in primary neocortical neuron cultures, we used cleaved caspase-3 immunolabeling to detect neuronal apoptosis. As shown in Figure 5(a), immunolabeling of cleaved caspase-3 was only detected in the t-BHP group. NSC-CM and Ly-NSC-CM significantly reduced cleaved caspase-3 immunolabeling. Based on the relative proportion of cleaved caspase-3-positive cells (% of total cells), the percentage of cleaved caspase-3-positive cells was significantly increased compared to the controls, to 43% after 10 *μ*M t-BHP exposure (*P* < 0.05; Figure 5(b)); treatment with NSC-CM and Ly-NSC-CM significantly attenuated the increase, to 28% and 15%, respectively (*P* < 0.05; Figure 5(b)). Ly-NSC-CM was significantly more effective than NSC-CM at attenuating the increase (*P* < 0.05; Figure 5(b)).

We then investigated the effect of lycopene on t-BHP-induced apoptosis in cultured hippocampal neurons using the TUNEL assay (Figure 5(c)). Numerous TUNEL-positive cells (green) were observed in t-BHP-treated cultures (47% of neurons), and significantly fewer were observed in control cultures (5%; *P* < 0.05; Figure 5(d)). Preincubation with NSC-CM and Ly-NSC-CM significantly reduced the percentages of TUNEL-positive cells to 29% and 14%, respectively (*P* < 0.05; Figure 5(d)); furthermore, Ly-NSC-CM reduced the percentage of TUNEL-positive cells significantly more than NSC-CM (*P* < 0.05; Figure 5(d)). These results indicated that NSC secretions were antiapoptotic as well.

### 3.6. Ly-NSC-CM Prevented t-BHP-Induced Expression of Apoptosis-Mediated Proteins in Neocortical Neurons

**Figure 6:**
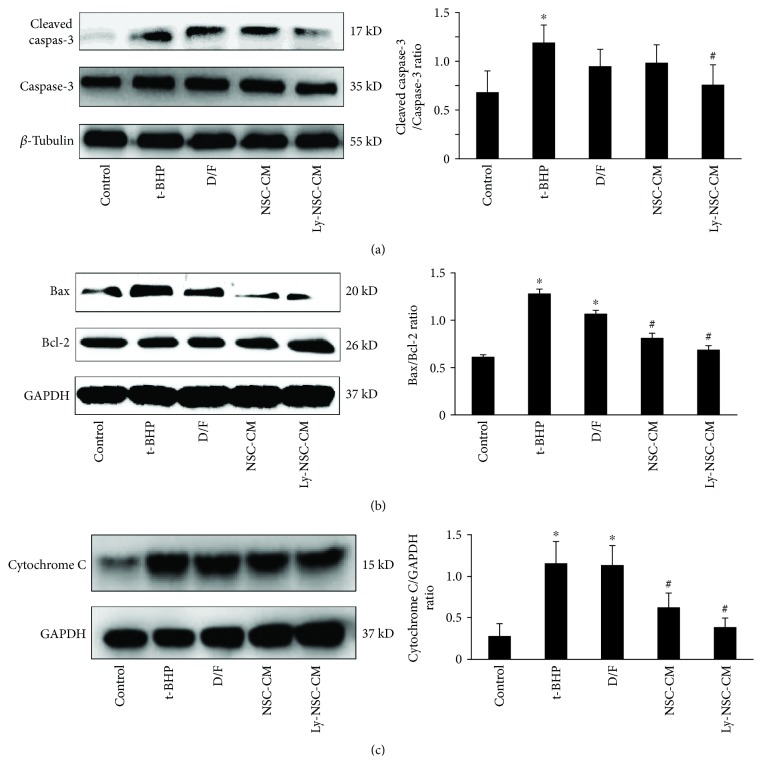
The expressions of Bax, Bcl-2, caspase-3, cleaved caspase-3, and cytochrome C in neurons. The expressions of Bax, Bcl-2, caspase-3, cleaved caspase-3, and cytochrome C were detected by Western blotting. GAPDH and *β-*tubulin were the housekeeping proteins. *n* = 3. Data are described as mean ± SEM. ^∗^*P* < 0.05 versus control group. ^#^*P* < 0.05 versus t-BHP group.

To further study the antiapoptotic role of Ly-NSC-CM in t-BHP-induced neuronal damage, we quantified cleaved caspase-3, intact caspase-3, Bax, Bcl-2, and cytochrome C protein expression by Western blotting. The results revealed that incubation with t-BHP caused a robust increase in Bax levels and a significant decrease in Bcl-2 levels, resulting in an approximately twofold increase in the Bax/Bcl-2 ratio. However, Ly-NSC-CM pretreatment significantly reversed this trend (*P* < 0.05; Figure 6(b)). Similarly, cleaved caspase-3 and cytochrome C were significantly reduced; the ratio of cleaved caspase-3 to intact caspase-3 significantly declined in the Ly-NSC-CM group relative to the t-BHP group (*P* < 0.05; Figures 6(a) and 6(c)). Thus, the effect of Ly-NSC-CM on t-BHP-induced apoptosis may at least partly be mediated by regulation of the mitochondrial apoptosis pathway.

### 3.7. Ly-NSC-CM Protects Neocortical Neurons from t-BHP-Induced Synaptic Damage

**Figure 7:**
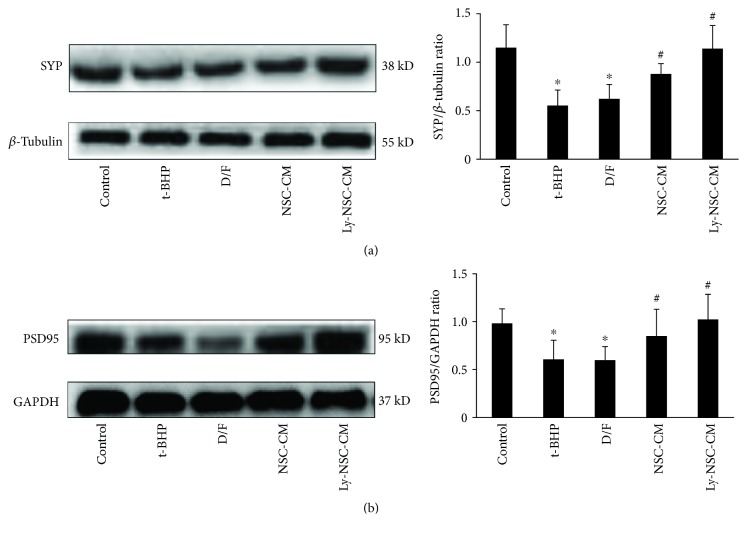
The expression of SYP and PSD95 in neurons after indicated treatment. The expressions of SYP and PSD95 were detected by Western blotting. GAPDH and *β-*tubulin was applied as the housekeeping protein, *n* = 3. Data was described as mean ± SEM. ^∗^*P* < 0.05 versus control group. ^#^*P* < 0.05 versus t-BHP group.

To determine the effect of Ly-NSC-CM pretreatment on t-BHP-induced neuronal damage on synaptic functional protein expression, Western blotting for the synaptic markers SYP and PSD95 was employed. The levels of SYP and PSD95 were significantly increased in the NSC-CM and Ly-NSC-CM groups relative to the t-BHP group (*P* < 0.05; Figures 7(a) and 7(b)). These data suggest that Ly-NSC-CM could effectively regulate the expression of synaptic proteins (SYP and PSD95) as a putative neuroprotective effect.

### 3.8. Ly-NSC-CM Protected Neocortical Neurons from t-BHP-Induced Oxidative Damage by Activating the PI3K/Akt Pathway

**Figure 8:**
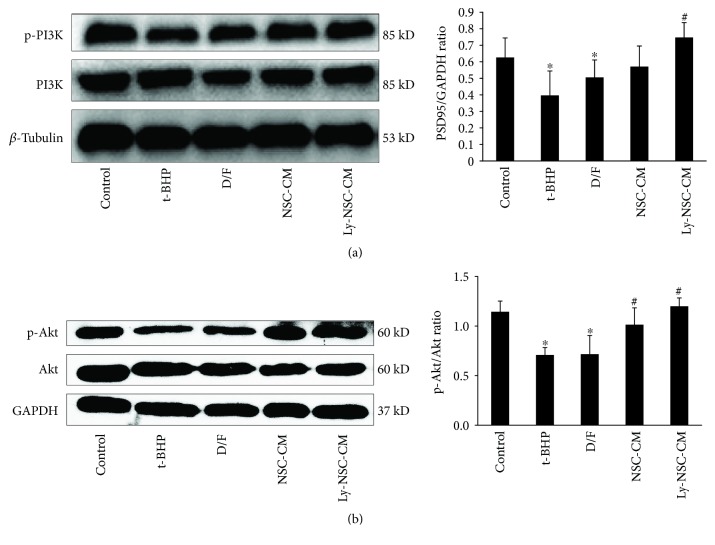
The activation of PI3K and Akt in neurons after indicated treatment. The changes of p-PI3K and p-Akt expression were compared with total PI3K and Akt. GAPDH and *β-*tubulin were applied as the housekeeping protein, *n* = 3. Data was described as mean ± SEM. ^∗^*P* < 0.05 versus control group. ^#^*P* < 0.05 versus t-BHP group.

Because the PI3K/Akt pathway is involved in antiapoptotic and prosynaptic regulation, we determined if these kinases were involved in the neuroprotection of Ly-NSC-CM by Western blotting for phosphorylated PI3K and Akt proteins. Both NSC-CM and Ly-NSC-CM significantly increased the expression of phosphorylated PI3K (*P* < 0.05; Figure 8(a)) and phosphorylated Akt (*P* < 0.05; Figure 8(b)); the Ly-NSC-CM group was significantly more effective in upregulating both proteins compared to the NSC-CM group.

## 4. Discussion

Oxidative stress is defined by an imbalance in the generation and clearance of reactive oxygen species (ROS), which can lead to oxidative damage to tissues and cells [27, 28]. ROS are mainly produced within the mitochondrial respiratory chain during energy metabolism. In the physiological state, the antioxidant defense system can effectively remove ROS, so that the generation and clearance of free radicals can maintain dynamic balance [29, 30]. However, in pathological states, a large amount of ROS overwhelms the body's antioxidant defense capacity, causing damage to membrane lipids, proteins, and nucleic acids along with other forms of oxidative damage, which in turn leads to a variety of diseases including neurodegenerative diseases, aging, and cancer [31–34].

In this study, we used t-BHP to induce oxidative injury experimentally. t-BHP is a stable exogenous oxidative agent that upon decomposition produces large amounts of free radicals that can induce multiple forms of oxidative stress in vitro, ultimately causing mitochondrial dysfunction and apoptosis [35–37]. It has been reported that t-BHP can cause neuronal oxidative stress and lead to upregulation of intracellular ROS, mitochondrial function damage, and eventually DNA damage and neuronal apoptosis [19, 20], phenomena which are consistent with oxidative stress injury mechanisms in AD. Therefore, we used t-BHP to establish a neuronal oxidative damage model in primary neonatal mouse cortical neurons.

We then assessed the ability of lycopene, a promising natural neuroprotectant, to induce the expression and excretion of therapeutically beneficial neurotrophic factors. We determined that lycopene at doses as low as 2 *μ*M could promote the secretion of NGF, BDNF, and VEGF in NSCs but had trivial effect on the secretion of EGF, with little to no secretion of bFGF and IGF-1 observed. Next, we sought to arrive at the optimal concentration of 10 *μ*M t-BHP to model injury. According to the results of repeated experiments and the relevant literature, 10 *μ*M t-BHP was decided on as the best concentration and 24 hours as the best t-BHP exposure time to assess cell death and damage, with neuron viability of about 50% observed in this protocol [23].

Having arrived at means for lycopene-mediated neurotrophic enrichment of NSC media (i.e., Ly-NSC-CM) and a model system to asses neuronal cell death relevant to AD, we tested the neuroprotective potential of Ly-NSC-CM. Media were collected and added to 10 *μ*M t-BHP-treated neurons. Light microscopy analysis showed that synapses were obviously ruptured or deteriorating, and neuronal nuclei were translucent in the 10 *μ*M t-BHP group, indicating synaptic damage and cell death. On the other hand, the Ly-NSC-CM group showed significantly decreased synaptic injury, with significant declines in synaptic deterioration, and the neuronal morphology was close to the control group. This suggests that Ly-NSC-CM may have protective effects on neurons and synapses and may also promote repair in neuronal synaptic injury. In accordance with these findings, protein levels of SYP and PSD95 in the Ly-NSC-CM group were obviously higher than those in the t-BHP group. Taken together, the data indicate that Ly-NSC-CM can effectively antagonize synaptic damage induced by t-BHP-induced oxidative stress, and this may be related to the regulation of synaptic-related protein expression. Furthermore, Ly-NSC-CM protected against cell death. Thus, Ly-NSC-CM is protective against neuronal damage at both the synaptic and whole-cell levels.

We then further tested the ability of t-BHP to stimulate the neuronal production of large amounts of ROS and the ability of conditioned media to lower ROS levels by flow cytometry analysis of ΔΨm. NSC-CM and Ly-NSC-CM both significantly reduced the level of ROS, thereby inhibiting t-BHP-induced neuron oxidative damage. However, Ly-NSC-CM had a better effect than NSC-CM on the restoration of normal ΔΨm in t-BHP-induced neuronal damage.

Next, we assessed protection against apoptosis, including analysis of effects along the apoptotic pathway. The expression of cleaved caspase-3 was measured by immunofluorescence, with the Ly-NSC-CM group showing significantly lower levels than the t-BHP group. Thus, Ly-NSC-CM prevented the activation of caspase-3 protein, suggesting the prevention of apoptosis. TUNEL assays further confirmed the antiapoptotic effect of Ly-NSC-CM. To further explore antiapoptotic mechanisms, the expressions of Bax, Bcl-2, caspase-3, cleaved caspase-3, and cytochrome C were detected by Western blot. The levels of Bax, cytochrome C, and cleaved caspase-3 in the Ly-NSC-CM group were obviously lower than those in the t-BHP group, while expression of the antiapoptotic protein Bcl-2 was significantly upregulated. Therefore, Ly-NSC-CM effectively antagonized t-BHP-induced neuronal oxidative damage and inhibited neuronal apoptosis. This might be related to direct regulation of apoptosis-related proteins. However, the targets and mechanisms of Ly-NSC-CM in regulating the expression of apoptosis-related proteins and synaptic-related proteins remained to be further explored.

Our study showed that the phosphorylation of PI3K and Akt protein in Ly-NSC-CM group was significantly upregulated. Studies had shown that PI3K/Akt signaling pathway is a key regulator of cell proliferation, differentiation, apoptosis, and aging. PI3K is activated by tyrosine kinase receptors, nontyrosine kinase receptors, and extracellular signals, such as insulin receptors. Akt activation is regulated by PI3K, which affects cell apoptosis in a variety of ways [38, 39]. It had been reported that activated Akt regulated the proapoptotic effect of Bad by promoting the phosphorylation of Bad and allowing Bad to escape from the Bcl-2/Bcl-X complex. On the other hand, Akt promotes Bcl-2 antiapoptotic effects, as Bcl-2 is located on the mitochondrial membrane and is a PI3k/Akt downstream factor [40, 41]. Moreover, it has been found that after Akt activation, phosphorylated caspase-9 was inactivated at Ser196, further inhibiting the activation of caspase-3 and other downstream molecules in the mitochondrial apoptotic signaling cascade [42–45]. Further, the activation of Akt was found to enhance the activity of the Bcl-2 promoter by augmenting the activity of cyclic AMP-response element-binding protein (CREB) and inhibiting the release of apoptosis-related factors by mitochondria, such as cytochrome C and AIF. The activation of PI3K/Akt also provides an important molecular basis for the initiation of apoptosis in a variety of cells against oxidative stress, ischemia, hypoxia/reoxygenation, and A*β* toxicity [44, 46–48]. Furthermore, it has been shown that Akt can regulate the activity of glycogen synthase, promote neuronal survival and apoptosis, reduce neurofibrillary tangles, and repair neuronal synaptic injury [49]. It has also been reported that the activation of PI3K/Akt pathway is associated with synaptic formation and long-term memory through long-term potentiation [50]. Taken together with these previous findings, upregulation of the PI3K/Akt by Ly-NSC-CM represents a possible mechanism for protection against neuronal oxidative damage induced by t-BHP.

## 5. Conclusions

From a broad perspective, in most cases, Ly-NSC-CM was significantly more beneficial than NSC-CM. These data suggest that preconditioning NSCs with lycopene could reinforce the antioxidant capacity and enhance growth factor secretion of NSCs. Collectively, our data are the first to demonstrate that a conditioned medium treated with the known neuroprotectant lycopene (Ly-NSC-CM) is sufficient to inhibit oxidative damage to primary cortical neurons induced by the oxidant t-BHP, most likely through the PI3K/Akt pathway.

There are, however, some limitations to our study. Firstly, we evaluated the neuroprotective effects of NSC-conditioned media, but not the potential anti-inflammatory effects and influence on A*β* oligomers. Secondly, we did not explore issues such as redox signaling and disruption of mitochondrial function that should also be disturbed by t-BHP. Finally, the study was limited to in vitro experiments, though future studies are focused on the in vivo effects. Nevertheless, our findings provide a new theoretical and experimental basis for the prevention and treatment of oxidative stress-related AD lesions.
